# Real world safety of methoxyflurane analgesia in the emergency setting: a comparative hybrid prospective-retrospective post-authorisation safety study

**DOI:** 10.1186/s12873-023-00862-2

**Published:** 2023-08-30

**Authors:** Nawab Qizilbash, Himanshu Kataria, Heather Jarman, Ben Bloom, Michelle Bradney, Maggie Oh, Sue Anne Yee, Ana Roncero, Ignacio Mendez, Stuart Pocock

**Affiliations:** 1 OXON Epidemiology, London, UK; 2 London School of Hygiene & Tropical Medicine, London, UK; 3 St Helens and Knowsley NHS Foundation Trust (Whiston Hospital), Prescot, UK; 4 St Georges Hospital NHS Foundation Trust, London, UK; 5 Barts Health NHS Trust, London, UK; 6 Medical Developments International Limited, Victoria, Australia

**Keywords:** Analgesia, Emergency department, Hepatotoxicity, Nephrotoxicity, Safety

## Abstract

**Background:**

Low-dose analgesic methoxyflurane (Penthrox^®^) was approved in Europe for emergency relief of moderate to severe pain in conscious adults with trauma in 2015. A comparative post-authorisation safety study (PASS) was conducted to assess the risk of hepatotoxicity and nephrotoxicity with methoxyflurane during routine clinical practice.

**Methods:**

This was a comparative hybrid prospective-retrospective cohort study. The comparative cohorts consisted of adults who were given methoxyflurane (methoxyflurane cohort) or another analgesic (concurrent cohort) routinely used for moderate to severe trauma and associated pain in the emergency setting (ambulance and Emergency Department) in the UK between December 2016 and November 2018. Hepatic and renal events were captured in the ensuing 12 weeks. A blinded clinical adjudication committee assessed events. A historical comparator cohort (non-concurrent cohort) was identified from patients with fractures in the English Hospital Episode Statistics (HES) accident and emergency database from November 2013 and November 2015 (before commercial launch of methoxyflurane). Hepatic and renal events were captured in the ensuing 12 weeks via linkage with the Clinical Practice Research Datalink (CPRD) and HES hospital admissions databases.

**Results:**

Overall, 1,236, 1,101 and 45,112 patients were analysed in the methoxyflurane, concurrent and non-concurrent comparator cohorts respectively. There was no significant difference in hepatic events between the methoxyflurane and concurrent cohorts (1.9% vs. 3.0%, P = 0.079) or between the methoxyflurane and non-concurrent cohorts (1.9% vs. 2.5%, P = 0.192). Renal events were significantly less common in the methoxyflurane cohort than in the concurrent cohort (2.3% vs. 5.6%, P < 0.001). For methoxyflurane versus non-concurrent cohort the lower occurrence of renal events (2.3% vs. 3.2%, P = 0.070) was not statistically significant. Multivariable adjustment did not change these associations.

**Conclusions:**

Methoxyflurane administration was not associated with an increased risk of hepatotoxicity or nephrotoxicity compared with other routinely administered analgesics and was associated with a reduced risk of nephrotoxicity compared with other routinely administered analgesics.

**Trial registration:**

Study registered in the EU PAS Register (ENCEPP/SDPP/13040).

**Supplementary Information:**

The online version contains supplementary material available at 10.1186/s12873-023-00862-2.

## Background

Methoxyflurane belongs to the fluorinated hydrocarbon group of volatile anaesthetics. Methoxyflurane (Penthrane®) was used as an inhalation anaesthetic during the 1960s, until discontinuation owing to reports of dose-related renal tubular damage at high anaesthetic doses [1, 2]. Hepatotoxicity resulting from high anaesthetic doses of methoxyflurane was also well described [3], where reported cases suggested an association with repeated exposure. In low doses, methoxyflurane has analgesic properties and has been widely used in Australia since 1975 in Ambulance Services, Emergency Departments (EDs), Defence Forces, and sporting fields. Methoxyflurane (Penthrox®) was approved in Europe in 2015 for the emergency relief of moderate to severe pain in conscious adults with trauma and associated pain [4]. The approval was largely based on the STOP! randomised controlled trial (RCT) [5].

Methoxyflurane is self-administered by the patient under supervision using a handheld inhaler, the “green whistle”, which provides up to 1 h of pain relief from a 3 mL dose if inhaled intermittently, or 20–25 min with continuous use [4, 6]. The maximum recommended dose is 6 mL (two 3 mL vials) in 24 h while administration on consecutive days is not recommended, and the total dose in a week should not exceed 15 mL [4]. A growing literature supports the efficacy and safety of methoxyflurane analgesia in the emergency setting and, where approved, for procedural analgesia [7–10], with over 8 million doses administered from 1975 to date. Mild adverse events such as nausea, dizziness, headache, dry mouth, and somnolence, are usually brief and self-limiting [5].

Hepatotoxicity is rare at low analgesic doses [11]. Acute hepatitis was described in a patient following repeated weekly exposure to methoxyflurane as procedural analgesia; it resolved within 4 weeks [12]. No evidence of hepatotoxicity or nephrotoxicity with methoxyflurane for analgesia has arisen from clinical trial data, although the duration of follow-up is limited, and the number of hepatic and renal events reported in routine pharmacovigilance are too few to draw reliable conclusions [5, 13–15]. A retrospective comparative observational study with a follow-up up to 14 years, showed no increased risk of hepatic or renal disease in patients given low-dose analgesic methoxyflurane [16].

Brief administration of methoxyflurane for analgesia in the emergency setting, and the possible delayed onset of any hepatotoxicity, means that routine pharmacovigilance may not be adequate to assess hepatotoxicity and nephrotoxicity. Therefore, the UK Medicines and Healthcare products Regulatory Agency (MHRA) requested a post authorisation safety study (PASS) to assess the risk of hepatotoxicity and nephrotoxicity with methoxyflurane analgesia (Penthrox®).

## Methods

### Objectives

The objectives were to assess the risk of hepatotoxicity and nephrotoxicity associated with administration of methoxyflurane for analgesia during routine clinical practice in the emergency setting (ambulance and EDs) in the UK. Off-label use, overdosage, and use in patients with a history of drug or alcohol abuse were assessed as exploratory objectives. Use in patients who presented with crush injury, heavy bleeding, low blood pressure or diabetes, or treated with contrast media or sevoflurane anaesthesia following methoxyflurane administration was also assessed.

### Study design

This PASS used a hybrid comparative prospective-retrospective study design. The primary data collection for the prospective comparative cohorts consisted of a methoxyflurane cohort that received methoxyflurane and a concurrent cohort that received other routinely administered analgesics. Patients were requested to participate in the study only after administration of methoxyflurane, to ensure the study did not influence the choice of analgesic. To increase statistical power, a retrospective non-concurrent cohort was identified from the English Hospital Episode Statistics (HES) accident and emergency (A&E) database and linked to routinely collected data by general practitioners in the Clinical Practice Research Datalink (CPRD) database and hospital admissions in the HES Admitted Patient Care (APC) database. The study protocol was approved by the MHRA and the study was registered in the EU PAS Register (ENCEPP/SDPP/13040).

### Prospective study

Primary data collection for the prospective comparative cohort study was conducted at 10 UK EDs from December 2016 to November 2018. Patients aged ≥ 18 years who were given methoxyflurane in the ambulance and/or ED were enrolled in the methoxyflurane cohort. Patients aged ≥ 18 years with trauma and associated pain who were given nitrous oxide, non-steroidal anti-inflammatory drugs (NSAIDs), opiates, or ketamine in the ambulance and/or ED were enrolled in the concurrent cohort.

Patients were followed for 12 weeks from the index date (date of the first dose of methoxyflurane or control analgesics) to identify hepatic and renal events via contact with the GP and patient (or nominated surrogate). When a hepatic or renal event was newly identified in the 12 weeks after administration of methoxyflurane or control analgesics, the local ED study team obtained the hospital records. Additional information on patient characteristics, medical history and risk factors, reasons for attending ED, medications received, contraindications to methoxyflurane, use of potentially hepatotoxic drugs before and during ED admission, admission/discharge, and vital status were recorded while the patient was in ED and from GP/patient/surrogate/hospital records.

### Retrospective study

The non-concurrent cohort was patients with fractures in the HES A&E database who were linked to CPRD, a database of anonymised electronic health records collected from general practitioners (GPs) in the UK and the HES-APC databases. Patients aged ≥ 18 years, with a record of A&E attendance with a fracture in the 24-month period prior to the launch date of Penthrox® in the UK, who were registered with the CPRD GP for at least 12 months before the index date (date of first admission of the patient to ED) were included in the non-concurrent cohort. Fracture was used as a proxy for trauma and associated use of analgesics.

Data on baseline patient characteristics, medical history and potential risk factors for hepatotoxicity and nephrotoxicity in the 12 months before the index date, reasons for attending ED, and vital status were collected via the patient’s electronic medical record in the HES A&E database and CPRD. Read codes and ICD-10 codes were used to identify hepatic and renal events that occurred within 12 weeks after the index date or were censored for the earliest of death, date of transfer out of the practice, or last practice collection date. Laboratory tests were also used to identify hepatic and renal events.

### Outcome measures

The primary and secondary endpoints were hepatic and renal events, respectively, in the 12 weeks after the index date. The criteria for hepatic and renal events are in Supplemental Appendix Table S1. For the prospective cohorts, a blinded adjudication committee (comprising two independent physicians with experience of drug-induced liver injury (JD and JA), and two independent physicians with experience of drug-induced renal injury (CW and JA)) confirmed the existence of a new event or worsening of a pre-existing condition from the hepatic and renal events notified. Events in the non-concurrent cohort were defined as those where the patient did not have a record of a verified disease/condition/abnormality affecting the liver or the kidney before the date of ED attendance for the fracture.

### Statistical analyses

The cumulative incidence of events is presented, and comparisons were made using Pearson’s chi-square or Fisher’s Exact Test. Student’s t-test and ANOVA were used to compare continuous variables. Incidence rates (cases/patient-month) for hepatic and renal events were estimated using Poisson regression. Confounding factors associated with hepatic and renal events were analysed and adjusted for with multivariable logistic regression. All statistical tests were two-sided and statistical significance was considered for P < 0.05 [17].

Data were analysed using SAS v9.4 and SAS Enterprise Guide v7.1.

### Sample size

A total of 1,250 patients in the methoxyflurane cohort was considered feasible within 2 years of the start of data collection to comply with regulatory obligations. Using the “Rule of Threes” if no hepatic events were observed, there would be 95% confidence that the real rate of hepatic events was below 2.4 per 1,000 patients (or 1/417). The “Rule of Threes” argued that if no case was seen in a sample of N patients, then the upper limit of the 95%CI is 3/N [18]. However, a few hepatic events were expected to occur in this population (N = 16), thus, a concurrent control cohort of a similar number of patients was included to test signals that breached this threshold. The statistical power was further improved by evaluating a non-concurrent cohort. Assuming that 0.5% of control patients experience hepatic events, with a non-inferiority ratio limit of 1.05, a sample size of 1,250 patients in the methoxyflurane cohort and 10,000 patients in the non-concurrent cohort would provide 80% power.

## Results

### Patients

**Fig. 1:**
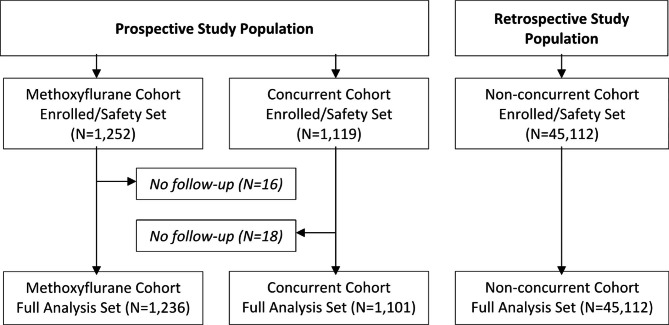
Study Population and Datasets Flow Chart

A total of 1,236, 1,101 and 45,112 patients were analysed in the methoxyflurane, concurrent, and non-concurrent cohorts respectively (Fig. 1). In the methoxyflurane cohort, methoxyflurane was administered to 98.7% of patients in ED, 1.1% in the ambulance, and 0.24% in both settings. Further analgesics (in addition to the index analgesic) were given to 79.3% (N = 979) of the methoxyflurane cohort and 48.4% (N = 533) of the concurrent cohort; none of the concurrent cohort received methoxyflurane. Usage of additional analgesics was higher in the concurrent cohort than in the methoxyflurane cohort, being statistically significant for NSAIDs, opioids and other drugs. It is not possible to determine the reason for this difference in further analgesic use because the study was not designed to assess effectiveness of pain relief of methoxyflurane. More patients in the methoxyflurane cohort (46.2%) than the concurrent cohort (35.3%) and non-concurrent cohort (11.2%) were admitted as inpatients from ED.

Patient characteristics were generally well balanced across the methoxyflurane and concurrent cohorts (Table 1). Few patients (< 1.5%) had been exposed to fluorinated anaesthetics in the 12 weeks before the index date, of whom none had a record of liver damage after exposure. Use of known hepatotoxic drugs within 12 weeks before the index date was higher in the non-concurrent cohort (38.3%) than the methoxyflurane (10.8%) and concurrent (12.2%) cohorts. Deaths during follow-up occurred in 0.4% (5/1,236), 0.4% (4/1,101), and 1.2% (559/45,112) of patients in the methoxyflurane, concurrent, and non-concurrent cohorts. No deaths in the methoxyflurane cohort were considered to be related to methoxyflurane or hepatic/renal disease.

**Table 1:** Patient Characteristics by Cohort (Full Analysis Set)

Characteristic	Methoxyflurane Cohort (N = 1236)	Concurrent Cohort (N = 1101)	Non-concurrent Cohort (N = 45112)
Age (years)			
Mean (SD)	46.0 (17.80)	46.5 (19.68)	50.9 (21.10)
Median (range)	45.0 (18–93)	44.0 (18–97)	50.0 (18–112)
> 75 [*n* (%)]	80 (6.47)	118 (10.72)	7129 (15.80)
Gender [*n* (%)]			
Female	550 (44.50)	505 (45.87)	23633 (52.39)
Medical history, [*n* (%)]			
Genetically susceptible to malignant hyperthermia	2 (0.16)	1 (0.09)	NC
History of severe adverse reactions	38 (3.07)	51 (4.63)	NC
History of drug abuse	21 (1.70)	19 (1.73)	182 (0.40)
History of alcohol abuse	29 (2.35)	34 (3.09)	933 (2.07)
Comorbidities in 12 weeks before index date, [*n* (%)]			
Viral hepatitis	4 (0.32)	0	9 (0.02)
Jaundice	1 (0.08)	2 (0.18)	6 (0.01)
LFT abnormal	21 (1.70)	26 (2.36)	1578 (3.50)
Chronic liver disease	5 (0.40)	4 (0.36)	75 (0.17)
Cholelithiasis	0	2 (0.18)	0
Other liver pathology	4 (0.32)	4 (0.36)	131 (0.29)
Renal impairment/failure	18 (1.46)	33 (3.00)	969 (2.15)
Other renal condition	25 (2.02)	28 (2.54)	107 (0.24)
Malignant neoplasm	17 (1.38)	9 (0.82)	1013 (2.25)
Congestive heart failure	4 (0.32)	11 (1.00)	240 (0.53)
Diabetes	57 (4.61)	60 (5.45)	1409 (3.12)
Obesity (BMI > 30)	51 (4.13)	57 (5.18)	4633 (10.27)
Presenting characteristics, [*n* (%)]			
Trauma	1213 (98.14)	1077 (97.82)	NC
Musculoskeletal condition	468 (37.86)	404 (36.69)	NC
Crush injury	55 (4.45)	57 (5.18)	NC
Head injury	34 (2.75)	58 (5.27)	NC
Altered level of consciousness	17 (1.38)	24 (2.18)	NC
Heavy bleeding	14 (1.13)	13 (1.18)	NC
Loss of consciousness	11 (0.89)	9 (0.82)	NC
Acute abdominal condition	1 (0.08)	2 (0.18)	NC
Clinically relevant hypotension	1 (0.08)	1 (0.09)	NC
Clinically evident respiratory depression	1 (0.08)	1 (0.09)	NC
Cardiac disorder	0	2 (0.18)	NC
Renal injury	0	1 (0.09)	NC
Medications received within 12 weeks before index date, [*n* (%)]			
Fluorinated anaesthetics	18 (1.46)	5 (0.45)	NC
Known hepatotoxic drugs	133 (10.76)	134 (12.17)	17260 (38.26)
Other additional analgesics used at the index date, [*n* (%)]			
Paracetamol	339 (68.5)	333 (74.2)	NC
codeine and paracetamol	21 (4.2)	32 (7.1)	NC
Lidocaine	35 (7.1)	16 (3.6)	NC
Dihydrocodeine and paracetamol	19 (3.8)	12 (2.7)	NC
Propofol	20 (4.0)	7 (1.6)	NC
Codeine	9 (1.8)	15 (3.3)	NC
Midazolam	14 (2.8)	10 (2.2)	NC
Fentanyl	11 (2.2)	6 (1.3)	NC
Diazepam	6 (1.2)	8 (1.8)	NC
Levobupivacaine	5 (1.0)	3 (0.7)	NC
Bupivacaine	5 (1.0)	1 (0.2)	NC
Ibuprofen	2 (0.4)	3 (0.7)	NC
Local anaesthetic	2 (0.4)	0 (0.0)	NC
Morphine	2 (0.4)	0 (0.0)	NC
Amoxicillin and clavulanic	1 (0.2)	0 (0.0)	NC
Dihydrocodeine	0 (0.0)	1 (0.2)	NC
Gabapentin	1 (0.2)	0 (0.0)	NC
Pethidine	1 (0.2)	0 (0.0)	NC
Pregabalin	0 (0.0)	1 (0.2)	NC
Prilocaine	0 (0.0)	1 (0.2)	NC

BMI = body mass index; LFT = liver function test; NC = not collected

### Hepatic events

There were 23, 33 and 1,112 hepatic events and 20, 23 and 287 confirmed hepatic events in the methoxyflurane, concurrent and non-concurrent cohorts respectively. There was no statistically significant difference between the methoxyflurane and concurrent cohorts in the cumulative incidence of all hepatic events [1.9% (23/1,236, 95% CI: 1.1, 2.6) vs. 3.0% (33/1,101, 95% CI: 2.0, 4.0), P = 0.079] or of confirmed events [1.6% (20/1,236, 95% CI: 0.9, 2.3) vs. 2.1% (23/1,101, 95% CI: 1.2, 2.9), p = 0.442] (Table 2). When comparing methoxyflurane and non-concurrent cohorts, there was no statistically significant difference in the incidence of all hepatic events [1.9% (23/1,236, 95% CI: 1.1, 2.6) vs. 2.5% (1,112/45,112, 95% CI: 2.3, 2.6), P = 0.192]; however, the incidence of confirmed hepatic events was significantly higher in the methoxyflurane cohort [1.6% (20/1,236, 95% CI: 0.9, 2.3) vs. 0.6% (287/45,112, 95% CI: 0.6, 0.7), P < 0.001]. Multiple logistic regression adjusting for potential confounders (age, sex, history of liver disease or cholelithiasis, alcohol or drug abuse or fluorinated anaesthetic administration, history of previous known hepatotoxic drugs, malignant neoplasm, congestive heart failure, obesity, and additional analgesics in ED) did not significantly affect these results. A description of confirmed hepatic events is in Supplemental Appendix Table S2.

**Table 2:** Hepatic Events During 12-Week Follow-up Period by Cohort (Full Analysis Set)

Variable	Methoxyflurane Cohort (1) (N = 1236)	Concurrent Cohort (2) (N = 1101)	Non-concurrent Cohort (3) (N = 45112)	p-value (1) vs. (2)	p-value (1) vs. (3)
**All Hepatic Events**					
No. of patients with events	23	33	1112	-	-
% of patients (95% CI)	1.9 (1.1, 2.6)	3.0 (2.0, 4.0)	2.5 (2.3, 2.6)	0.079	0.192
**Incidence Rate for All Hepatic Events**					
No. of valid patients^a^	1222	1048	45112	-	-
No. of patients with events (%)	22 (1.80)	32 (3.05)	1112 (2.46)	-	-
Incidence rate (patient-month) (95% CI)	6.62 (4.15, 10.03)	11.34 (7.75, 16.00)	9.16 (8.63, 9.72)	0.052	0.131
Incidence rate ratio^b^ (95% CI)	-	1.71 (0.99, 2.95)	1.40 (0.91, 2.11)	-	-
**Confirmed Hepatic Events**					
No. of patients with events	20	23	287	-	-
% of patients (95% CI)	1.6 (0.9, 2.3)	2.1 (1.2, 2.9)	0.6 (0.6, 0.7)	0.442	< 0.001
**Incidence Rate for Confirmed Hepatic Events**					
No. of valid patients^a^	1223	1049	45112	-	-
No. of patients with events (%)	20 (1.64)	23 (2.19)	287 (0.64)	-	-
Incidence rate (patient-month) (95% CI)	6.01 (3.67, 9.28)	8.08 (5.12, 12.13)	2.33 (2.07, 2.62)	0.332	< 0.001
Incidence rate ratio^b^ (95% CI)	-	1.34 (0.74, 2.45)	0.39 (0.25, 0.61)	-	-

CI = confidence interval

^a^ Patients with complete follow up

^b^ Methoxyflurane cohort is reference category

**Fig. 2:**
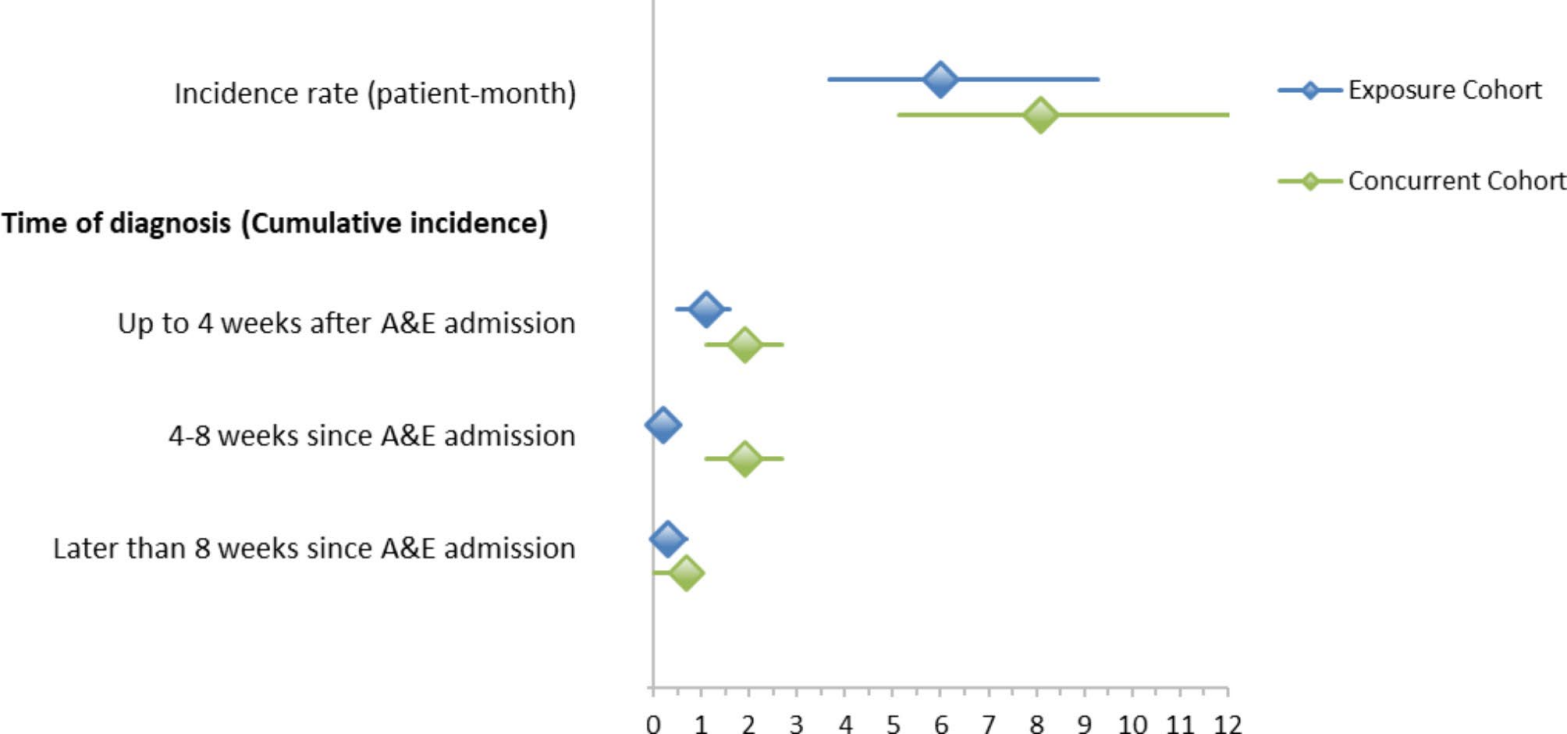
Confirmed Hepatic Events - Incidence Rates, and Cumulative Incidence Rates overall and by Time Periods) Methoxyflurane versus concurrent cohorts X-axis represents the percentage of confirmed hepatic events Y-axis represents weeks

Most hepatic events occurred within 4 weeks of the index date (Fig. 2). In the methoxyflurane/concurrent cohort comparison independent predictors associated with confirmed hepatic events were age (odds ratio [OR]: 1.0; 95% CI: 1.0, 1.1; P < 0.001), history of alcohol abuse (OR: 7.6; 95% CI: 3.2, 18.2; P < 0.001), use of additional analgesics in ED (OR: 2.5; 95% CI: 1.1, 5.7; P = 0.026) and previous use of potentially hepatotoxic drugs (OR: 3.3; 95% CI: 1.7, 6.8; P < 0.001), but not with methoxyflurane (OR: 0.7; 95% CI: 0.4, 1.4; P = 0.296). The same independent predictors, a history of alcohol abuse (OR: 12.5; 95% CI: 4.0, 39.0; P < 0.001) and previous use of known potentially hepatic drugs (OR: 3.5; 95% CI: 1.3, 9.5; P = 0.013), were also found for the methoxyflurane/non-concurrent cohort comparison.

### Renal events

There were 28, 62 and 1,450 renal events and 9, 29, and 1,106 confirmed renal events in the methoxyflurane, concurrent and non-concurrent cohorts, respectively. The cumulative incidence of all renal events was statistically significantly lower in the methoxyflurane cohort than in the concurrent cohort [2.3% (28/1,236, 95% CI: 1.4, 3.1) vs. 5.6% (62/1,101, 95% CI: 4.3, 7.0), P < 0.001] and also for confirmed events [0.7% (9/1,236, 95% CI: 0.3, 1.2) vs. 2.6% (29/1,101, 95% CI: 1.7, 3.6), P < 0.001]. For methoxyflurane versus non-concurrent cohorts there was no significant difference for all renal events [2.3% (28/1236, 95% CI: 1.4, 3.1) vs. 3.2% (1450/45,112, 95% CI: 3.1, 3.4), P = 0.070] and a significant difference for confirmed events [0.7% (9/1,236, 95% CI: 0.3, 1.2) vs. 2.5% (1,106/45,112, 95% CI: 2.3, 2.6), P < 0.001]. Multiple logistic regression adjusting for potential confounders (age, sex, history of renal disease or impairment, diabetes, alcohol or drug abuse or fluorinated anaesthetic administration, congestive heart failure, and additional analgesics in ED) did not significantly affect these results. Incidence rates of all renal events and confirmed renal events were also statistically significantly lower in the methoxyflurane cohort than in the concurrent and non-concurrent cohorts (Table 3). A description of the confirmed renal events is in Supplemental Appendix Table S3.

**Table 3:** Renal Events During 12-Week Follow-up Period by Cohort (Full Analysis Set)

Variable	Methoxyflurane Cohort (1) (N = 1236)	Concurrent Cohort (2) (N = 1101)	Non-concurrent Cohort (3) (N = 45112)	p-value (1) vs. (2)	p-value (1) vs. (3)
All Renal Events					
No. of patients with events	28	62	1450	-	-
% of patients (95% CI)	2.3 (1.4, 3.1)	5.6 (4.3, 7.0)	3.2 (3.1, 3.4)	< 0.001	0.070
**Incidence Rate for All Renal Events**					
No. of valid patients^a^	1221	1039	45112	-	-
No. of patients with events (%)	26 (2.13)	52 (5.00)	1450 (3.21)	-	-
Incidence rate (patient-month) (95% CI)	7.89 (5.15, 11.56)	18.76 (14.01, 24.61)	11.98 (11.37, 12.61)	< 0.001	0.034
Incidence rate ratio^b^ (95% CI)	-	2.38 (1.49, 3.81)	1.52 (1.03, 2.24)	-	-
**Confirmed Renal Events**					
No. of patients with events	9	29	1106		
% of patients (95% CI)	0.7 (0.3, 1.2)	2.6 (1.7, 3.6)	2.5 (2.3, 2.6)	< 0.001	< 0.001
**Incidence Rate for Confirmed Renal Events**					
No. of valid patients^a^	1223	1048	45112	-	-
No. of patients with events (%)	9 (0.74)	28 (2.67)	1106 (2.45)	-	-
Incidence rate (patient-month) (95% CI)	2.69 (1.23, 5.10)	9.84 (6.54, 14.23)	9.09 (8.56, 9.64)	0.004	< 0.001
Incidence rate ratio^b^ (95% CI)	-	3.66 (1.72, 7.56)	3.38 (1.75, 6.52)	-	-

CI = confidence interval

^a^ Patients with complete follow up

^b^ Methoxyflurane cohort is reference category

**Fig. 3:**
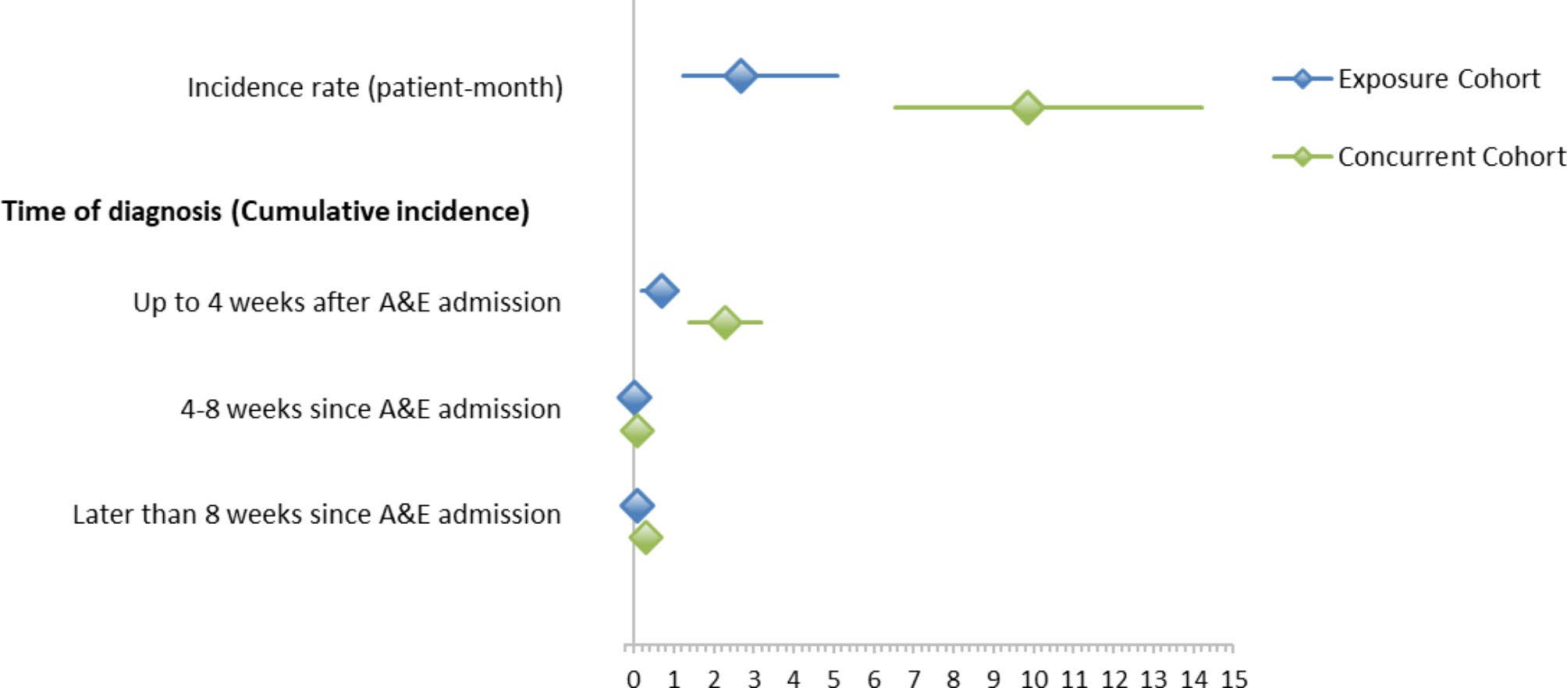
Confirmed Renal Events - Incidence Rates, and Cumulative Incidence Rates overall and by Time Periods Methoxyflurane versus concurrent cohorts X-axis represents the percentage of confirmed renal events Y-axis represents weeks

Most renal events occurred within 4 weeks of the index date (Fig. 3). Methoxyflurane was found to be an independent predictor associated with a lower risk of confirmed renal events in the methoxyflurane/concurrent cohort multivariable analyses (OR: 0.3; 95% CI: 0.1, 0.6; P = 0.002). Other predictors associated with confirmed renal events in the methoxyflurane -concurrent cohort comparison were age (OR: 1.1; 95% CI: 1.1, 1.1; P < 0.001), renal impairment/failure (OR: 4.7; 95% CI: 1.9, 11.9; P = 0.001), diabetes (OR: 3.8; 95% CI: 1.7, 8.8; P = 0.002) and use of additional analgesics in ED (OR: 3.0; 95% CI: 1.3, 7.0; P = 0.010). The same predictors, plus congestive heart failure (OR: 2.2, 95% CI: 1.5, 3.3; P < 0.001) were found for the methoxyflurane/non-concurrent cohort comparison.

### Exploratory outcomes

Off-label use of methoxyflurane was low [21 patients (1.7%, 95% CI: 1.0, 2.5) in the methoxyflurane cohort]. Individual contraindications were altered level of consciousness (81.0%, 17/21, 95% CI: 64.2, 97.8), malignant hyperthermia (9.5%, 2/21, 95% CI: 0.0, 22.1), hypersensitivity to methoxyflurane or any fluorinated anaesthetic (4.8%, 1/21, 95% CI: 0.0, 13.9), and clinically evident respiratory depression (4.8%, 1/21, 95% CI: 0.0, 13.9). Five patients (0.4%, 5/1,252) received methoxyflurane for musculoskeletal pain of non-traumatic origin. The proportion of patients with a history of drug or alcohol abuse was low (≤ 3.1%) in all three cohorts (Table 1). There were no cases of methoxyflurane overdose (administration of > 2 vials in 24 h in the ED).

It was impossible to assess whether there was an increased risk of hepatotoxicity and nephrotoxicity in methoxyflurane recipients who presented with crush injury, heavy bleeding, low blood pressure and diabetes, owing to the few hepatic (N = 20) and renal (N = 9) events in these patients.

The number of patients exposed to contrast media during follow-up was 37/1,236 (3.0%) and 40/1,101 (3.6%) in the methoxyflurane and concurrent cohorts respectively. Multivariable analysis revealed no excess risk of confirmed hepatic or renal events in patients given contrast media after methoxyflurane, adjusted for other baseline variables.

The number of patients exposed to sevoflurane after index treatment was 158/1236 (12.8%) and 80/1101 (7.3%) in the methoxyflurane and concurrent cohorts respectively. Multivariable analyses showed a non-statistically significant lower risk of confirmed hepatic and renal events with methoxyflurane and sevoflurane. Univariate analyses showed statistically significant lower risk of confirmed renal events, and non-statistically significant lower risk of confirmed hepatic events, with methoxyflurane and sevoflurane.

In the methoxyflurane cohort, 0.5% (6/1,234) of patients reported a non-hepatic or non-renal adverse event such as headache, nausea and dizziness.

## Discussion

This PASS shows no increased risk of hepatotoxicity or nephrotoxicity in patients given methoxyflurane compared with patients given other routine analgesics within 12 weeks of exposure. The results indicate a higher risk of nephrotoxicity in patients who were given other routine analgesics than in those given methoxyflurane.

The use of methoxyflurane followed the guidance in the Summary of Product Characteristics (SmPC), with little off-label use and no overdosage. A few patients with a history of alcohol abuse or of potentially hepatotoxic drugs were exposed to methoxyflurane but these factors were found independently associated with hepatic events and they are contraindicated in the SmPC. Contrast media, which was infrequently given after methoxyflurane, was not associated with a higher risk of hepatic or renal events.

An anomaly is the apparent increased risk of confirmed hepatic events comparing methoxyflurane with the non-concurrent cohort. The rates of events in the non-concurrent cohort should be similar to the non-methoxyflurane concurrent cohort to be considered comparable. Similar rates were found for all hepatic events in the non-methoxyflurane concurrent and non-concurrent cohorts, but they were markedly different for confirmed hepatic events. While 70% of “all hepatic events” were classified as “confirmed hepatic events” for the non-methoxyflurane concurrent cohort, 24% of “all hepatic events” were classified as “confirmed” events in the non-concurrent cohort. This discrepancy reflects differences in the presentation and indication of the concurrent cohort and non-concurrent cohort, the latter included only fractures and not all may have had moderate to severe pain requiring analgesic included in the study, as well as the way events were confirmed (see supplementary Table S1), although this explanation could not be tested. In the prospective cohorts, hepatic and renal events were confirmed by a blinded clinical adjudication committee, while in the non-concurrent cohort, by the absence of a prior record of hepatic or renal abnormalities. Results from analyses where there were large differences in the populations and nature of events between the historical cohort and the concurrent cohort should be viewed with caution. However, results from analyses where there were few differences in the populations and nature of events between the historical cohort and the concurrent cohort can be interpreted with more confidence. A proportionally greater reduction in ´confirmed´ hepatic events in the non-concurrent cohort was observed, compared to the reduction observed in ´confirmed´ renal events, but the reason for this difference could not be tested. Comparison with the non-concurrent cohort should be interpreted with caution.

The strengths of the study included its large size (more than the total number of patients in all previous randomised trials of methoxyflurane combined), the multiple methods to capture endpoints and its real-world setting for generalisability. The use of an independent blinded adjudication committee minimised bias in the assessment of endpoints. The non-concurrent cohort allowed for good statistical power to exclude small excess risks.

There are several limitations. The size of the prospective comparative study alone was underpowered to exclude modest excess relative risks of both hepatic and renal events. However, an adequately powered prospective comparative study would have taken many years to recruit. While the non-concurrent cohort was designed to overcome the issue of low statistical power, it was not exactly comparable to the concurrent prospective comparator cohort, because this cohort came from a hospital ED database which does not have data on the use of analgesics in ED and used fractures as a proxy for a population of patients with moderate to severe pain likely to be treated with analgesics. Lastly, confounding factors were adjusted for in multivariate analysis. However, as in any observational study, there remains the possibility of residual confounding.

## Conclusions

In conclusion, methoxyflurane administration was not associated with an increased risk of hepatotoxicity or nephrotoxicity compared with other routinely administered analgesics. Methoxyflurane administration was associated with a reduced risk of nephrotoxicity compared with other routinely administered analgesics.

### Electronic supplementary material

Below is the link to the electronic supplementary material.

**Supplementary Appendix: Table S1** Definition of hepatic events. **Table S2** Description of Confirmed Hepatic Events During 12-Week Follow-up Period by Cohort. **Table S3** Description of Confirmed Renal Events During 12-Week Follow-up Period by Cohort (Full Analysis Set)

## Data Availability

The retrospective data for this study were obtained from the UK CPRD and HES databases. CPRD data governance does not allow us to distribute patient data to other parties. Researchers can apply for data access upon reasonable request and with permission from Medical Development International Pty Limited / OXON Epidemiology (contact: Nawab Qizilbash MRCP(UK) DPhil(Oxon), Senior Clinical Epidemiologist, OXON Epidemiology, Calle Doctor Fleming 51, 28036 Madrid, Spain. Email: n.qizilbash@oxonepi.com). This study was approved by the Medicines and Healthcare products Regulatory Agency’s Independent Scientific Advisory Committee (protocol number 19_195). We registered our protocol in the European Union Electronic Register of Post-Authorisation Studies on April 2016 (protocol number EUPAS 13040).

## Declarations

To enable use of the retrospective data, the study was approved by the Medicines and Healthcare products Regulatory Agency’s Independent Scientific Advisory Committee (protocol number 19_195), and no separate ethical committee approval was required for use of this data. The protocol is available online.

The manuscript has been written in accordance with STROBE [19], Good Pharmacoepidemiology Practice [20] and Good Publication Practice guidelines [21].
