# Supplementary material for: Real world safety of methoxyflurane analgesia in the emergency setting: a comparative hybrid prospective-retrospective post-authorisation safety study
Source: BMC Emerg Med. 2023 Aug 30;23:100. doi: 10.1186/s12873-023-00862-2 (PMC10469512; doi:10.1186/s12873-023-00862-2)
Supplement: Supplementary file 1 — Supplementary Appendix: Table S1 Definition of hepatic events. Table S2 Description of Confirmed Hepatic Events During 12-Week Follow-up Period by Cohort. Table S3 Description of Confirmed Renal Events During 12-Week Follow-up Period by Cohort (Full Analysis Set) [file 12873_2023_862_MOESM1_ESM.docx]

**Supplementary Appendix**

**Table S1**

| ***Definition of hepatic events*** | |
| --- | --- |
| ***Prospective methoxyflurane and concurrent cohorts*** | 1) All hepatic events. Identification of all hepatic dysfunction events during the follow-up. Any patient where any of the following were recorded (YES):   - - Jaundice; hospitalisation with hepatic event or deranged liver function tests; any liver disease/ condition/ abnormality; or death with hepatic diagnosis.   - ALT or AST value ≥3 times the ULN (3xULN) (ULN = 56 and 40 international units per litre, respectively), ALP value ≥2 x ULN (ULN = 147 IU/L (international units per litre)), total bilirubin ≥2 x ULN (ULN = 20 µmol/L) or INR >1.5 (not due to anticoagulants)   - All patients with a positive response had a corresponding Safety Information Reporting form and a ‘Potential Hepatotoxicity Questionnaire’.   - Confirmed hepatic events. As the AC did not have sufficient clinical information to be able to reliably judge if a drug could be related to the hepatic event, a further outcome category was devised: confirmed hepatic cases. These were all cases in which the event was confirmed as being a true hepatic event, with or without clinical or biochemical evidence of a hepatic event before the index date. |
|  | 2) Hepatotoxic events. Validation of the hepatic dysfunction events as hepatotoxic events were performed by the AC as follows: All hepatic dysfunction events followed a validation process by the AC As a result of each assessment an Adjudication Committee form for hepatic events was completed. These forms validated the existence of a hepatic event and assessed if the event was compatible with having been potentially induced by a drug, and if that was the case, if it was compatible with a drug administered at the index date or if this aetiology could not be rejected. These two latter cases were the validated hepatotoxic events.  All assessments performed by the AC were blind to methoxyflurane or control group status. |
| ***Non-concurrent cohort*** | 1) Hepatic events: Identification of any of the following for a given patient during follow-up (patients may have had more than one of these recorded:   - - Any code for jaundice during follow-up, identified by Read codes in CPRD and ICD-10 codes in any of the diagnosis fields of a hospital admission in HES-APC.   - Any hepatic event identified by Read codes in CPRD   - Any hepatic event identified by ICD-10 codes in any of the diagnosis fields of a hospital admission in HES-APC   - Abnormal liver function test results. The definition of an abnormal liver function test result indicative of a hepatic dysfunction event was either an increase of ≥3x ULN for ALT or AST; ALP value ≥2 x ULN, total bilirubin ≥2 X ULN or INR >1.5 (not due to anticoagulants) |
|  | 2) Confirmed hepatic events: In addition to the presence of any of the above, the absence of all of the following were determined in order to confirm that the event was an incident (post-index date) record of a hepatic event:   - - Any Read code in CPRD or ICD-10 code in any of the diagnosis fields of a hospital admission in HES for jaundice or cholelithiasis in the 12 weeks prior to index date   - Any Read code in CPRD or ICD-10 code in any of the diagnosis fields of a hospital admission in HES for any other hepatic event in the 12 months prior to index date   - Abnormal liver function test result (as defined above) in the 12 weeks prior to index date   - History of drug or alcohol abuse |
| ***Definition of renal events*** | |
| ***Methoxyflurane and concurrent cohorts*** | 1) Identification of all renal dysfunction events during the follow-up: Any patient where a positive response (YES) could be found for any of the following:   - - Any renal disease/condition/abnormality; renal impairment/failure, pre-renal impairment/failure, hospitalisation with any data to suggest a renal event or impaired renal function tests, death with any data to suggest a renal event.   - Among patients with lab test results available: Serum creatinine value ≥26.5 µmol/L (≥ 0.3 mg/dL) OR 1.5-fold increase from the index date due to unknown cause OR estimated glomerular filtration rate (eGFR < 60mL/min/1.73 square metres).   All patients with a positive response had a corresponding Safety Information Reporting form and a ‘Potential Nephrotoxicity Questionnaire’ completed.  Confirmed renal events. As the AC did not have sufficient clinical information to be able to reliably judge if a drug could be related to the renal event, a further outcome category was devised: confirmed renal cases. These were all cases in which the event was confirmed as being a true renal event with or without clinical or biochemical evidence of a renal event before the index date. |
|  | 2) Validation of the renal dysfunction events as nephrotoxic events by the AC: All renal dysfunction events followed a validation process by the AC. As a result of each assessment, an Adjudication Committee form for renal events was completed. These forms validated the existence of a renal event and assessed if the event was compatible with having been potentially induced by a drug, and if that was the case, if it was compatible with a drug administered at the index date or if this aetiology could not be rejected. These two latter cases were the validated nephrotoxic events.  All assessments performed by the AC were blind to methoxyflurane or control group status. |
| ***Non-concurrent cohort*** | 1) Renal events: Identification of any of the following for a given patient during follow-up (patients may have had more than one of these but will only have one renal event recorded:   - - Any renal event identified by Read codes in CPRD   - Any renal event identified by ICD-10 codes in any of the diagnosis fields of a hospital admission in HES-APC   - Abnormal renal function test results. The definition of an abnormal renal function test result indicative of an adverse renal event is a change in serum creatinine value ≥26.5 µmol/L (≥ 0.3 mg/dL) OR 1.5-fold increase from a serum creatinine test conducted in the 12 weeks before the index date due to unknown cause OR eGFR <60mL/min/1.73 square metres (calculated using the CKD-EPI) where not provided and data were available for the calculation) |
|  | 2) Confirmed renal events: In addition to the presence of any of the above, the absence of all the following was required to confirm that the event was an incident (post-index date) record of a renal event:   - - Any Read code in CPRD or ICD-10 code in any of the diagnosis fields of a hospital admission in HES for renal event in the 12 months prior to index date   - Abnormal renal function test result (as defined above) in the 12 weeks prior to index date   - History of drug or alcohol abuse |

AC= Adjudication Committee; ALT= alanine transaminase; AST= aspartate aminotransferase; ALP= alkaline phosphatase; CPRD= Clinical Practice Research Datalink; CKD-EPI= Chronic Kidney Disease Epidemiology Collaboration; eGFR= estimated glomerular filtration rate; HES-APC= Hospital Episode Statistics – Admitted Patient Care; ICD-10= International Classification of Diseases; INR= international normalized ratio; ULN= upper limit of normality.

**Table S2** **Description of Confirmed Hepatic Events During 12-Week Follow-up Period by Cohort**

| **Variable** | **Methoxyflurane Cohort (1)**  **(N=1,236)** | **Concurrent Cohort (2)**  **(N=1,101)** | **Non-concurrent Cohort (3) (N=45,112)** | **p-value**  **(1) vs (2)** | **p-value**  **(1) vs (3)** |
| --- | --- | --- | --- | --- | --- |
| **Confirmed hepatic events [*n* (%)]** | **20 (1.62)** | **23 (2.09)** | **287 (0.64)** | **0.200** | **<0.001** |
| Type of event (n) |  |  |  |  |  |
| Cholestatic | 7 | 10 | - | 0.784 | - |
| Hepatocellular | 10 | 9 | - | - | - |
| Mixed | 3 | 4 | - | - | - |
| Severity of event (n) |  |  |  |  |  |
| Grade 1 | 10 | 10 | - | 0.117 | - |
| Grade 2 | 8 | 6 | - | - | - |
| Grade 3 | 1 | 7 | - | - | - |
| Grade 4 | 1 | 0 | - | - | - |

ALP=alkaline phosphatase; ALT=alanine aminotransferase; AST=aspartate aminotransferase; INR=international normalized ratio; LFT=liver function test; ULN=upper limit of normal (range).

Patients may have hepatic events in more than 1 category.

**Table S3** **Description of Confirmed Renal Events During 12-Week Follow-up Period by Cohort (Full Analysis Set)**

| **Variable** | **Methoxyflurane Cohort (1)**  **(N=1,236)** | **Concurrent Cohort (2)**  **(N=1,101)** | **Non-concurrent Cohort (3) (N=45,112)** | **p-value**  **(1) vs (2)** | **p-value**  **(1) vs (3)** |
| --- | --- | --- | --- | --- | --- |
| **Confirmed renal events [*n* (%)]** | **9 (0.73)** | **29 (2.63)** | **1106 (2.45)** | **0.249** | **<0.001** |
| Type of event (n) |  |  |  |  |  |
| Chronic kidney injury | 1 | 2 | - | 1.000 | - |
| Acute kidney injury | 8 | 26 | - | - | - |
| Grade 1 | 4 | 23 | - | - | - |
| Grade 2 | 3 | 1 | - | - | - |
| Grade 3 | 1 | 2 | - | - | - |
| Severity of event (n) |  |  |  |  |  |
| Grade 1 | 5 | 25 | - | - | - |
| Grade 2 | 3 | 1 | - | - | - |
| Grade 3 | 1 | 1 | - | - | - |
| Grade 4 | 0 | 1 | - | - | - |

GFR=glomerular filtration rate.

Patients may have renal events in more than 1 category.
